# Association between lung fluid levels estimated by remote dielectric sensing and lung ultrasound in patients with heart failure

**DOI:** 10.3389/fcvm.2026.1723377

**Published:** 2026-01-29

**Authors:** Shan Huang, Guangfeng Sun, Yulin Wu, Hongfei Jiang, Weiliang Zhu, Penglong Wu, Manxin Lin, Fanqi Meng

**Affiliations:** 1 Department of Cardiology, Xiamen Cardiovascular Hospital of Xiamen University, School of Medicine, Xiamen University, Xiamen, China; 2 Department of Emergency, Xiamen Cardiovascular Hospital of Xiamen University, School of Medicine, Xiamen University, Xiamen, China

**Keywords:** remote dielectric sensing, lung ultrasound, lung fluid evaluation, heart failure, relationship

## Abstract

**Background:**

Lung ultrasound (LUS) has been established as a standard modality for assessing pulmonary congestion in heart failure. Remote Dielectric Sensing (ReDS) represents a novel, rapid, non-invasive technology for quantifying pulmonary fluid content. However, the correlation between ReDS and LUS findings in heart failure patients remains undefined.

**Methods:**

In this prospective, single-center observational study from March 2024 to June 2024, patients with heart failure were consecutively enrolled and underwent ReDS and LUS examinations. We assessed the agreement between these two modalities in measurement of lung fluid.

**Results:**

Among 153 enrolled patients [median age 74 (60, 81) years; 92 male], median values were 32% (range 16%–59%) for ReDS measurements and 7 (range 0–32) for B-line count. 49 (32%) patients demonstrated significant pulmonary congestion on LUS. There was a significant positive correlation between ReDS values and sum of B-lines on LUS (*r* = 0.544, *p* < 0.001). In multivariate linear regression, ReDS levels (*β* = 0.569, *p* < 0.001) showed independent associations with the B-line count. At a cutoff of 34.5%, ReDS demonstrated the ability to identify pulmonary congestion on LUS, with an area under the curve of 0.748, sensitivity of 73.5%, and specificity of 70.2%.

**Conclusions:**

ReDS technology showed marked correlation with B-line counts and fair diagnostic accuracy in detecting pulmonary congestion on LUS, suggesting its potential utility for volume assessment in heart failure management.

**Trial registration:**

ChiCTR2400081719 in the Chinese Clinical Trial Registry.

## Introduction

Despite the remarkable advancement of both pharmacological and device-based therapies in heart failure (HF), the condition remains a formidable global health burden due to its escalating prevalence and persistently high mortality rates (1–3). The recognition and management of pulmonary congestion, which serves as a key pathophysiological hallmark and therapeutic target, are fundamental components in the care of patients with HF (4, 5). While lung ultrasound (LUS) has become an established point-of-care tool for detecting pulmonary congestion through the assessment of B-lines (6, 7) which contributes to both diagnostic evaluation and therapeutic guidance in HF (8–11), its practical application can be limited by factors such as the time required for examination, the need for patient disrobing, and gel use.

Remote dielectric sensing (ReDS) is a novel, noninvasive device that quantifies lung fluid levels as a percentage within a minute and can be used over the patient's garments (12). Previous validation studies have demonstrated significant correlations between ReDS measurements and both invasive hemodynamic parameters (pulmonary artery wedge pressure) and radiographic assessments (chest computed tomography) of lung water content (13, 14). Notably, recent clinical evidence positions ReDS as superior to conventional chest x-ray for grading mild pulmonary congestion (15).

However, the relationship between ReDS-derived lung fluid quantification and the well-validated LUS assessment is not well defined. Therefore, the primary objective of this study was to systematically evaluate the correlation between ReDS and LUS measurements in patients with HF, and to explore the clinical applicability of ReDS technology in this population.

## Methods

### Study design and population

In this prospective single-center observational study, all consecutive adult patients with heart failure, who were admitted to the cardiac intensive care unit (CICU) of Xiamen Cardiovascular Hospital of Xiamen University (Xiamen, China) from March 2024 to June 2024 were enrolled. The diagnosis of HF was established by the attending physician in accordance with the diagnostic algorithm for HF from 2021 ESC Guidelines (16), which was based on a combination of the following: (1) the presence of typical signs and symptoms (e.g., dyspnea, orthopnea, peripheral edema, pulmonary rales); (2) elevated levels of N-terminal pro-B-type natriuretic peptide (NT-proBNP > 125 pg/mL); and (3) objective evidence of cardiac structural or functional abnormality obtained from systematic echocardiography performed upon/before admission. Exclusion criteria comprised patients who were ineligible for ReDS testing, such as intra-aortic balloon pump (IABP) implantation, extracorporeal membrane oxygenation (ECMO), mechanical ventilation, body mass index (BMI) outside the range of 18–38 kg/m^2^, absence of the right lung, thoracic deformities, rib fractures, dextrocardia, or right-sided pacemakers; those with severe pulmonary diseases including chronic obstructive pulmonary disease, asthma, bronchiectasis, or lung tumors; individuals with significant pleural effusion, empyema, pneumothorax, hemothorax, or hemopneumothorax; and those who declined to participate.

Our work had been carried out in accordance with Declaration of Helsinki for experiments involving humans. This study has been approved by the Ethics Committee of Xiamen Cardiovascular Hospital of Xiamen University (2024YLK13) and registered in the Chinese Clinical Trial Registry (ChiCTR2400081719).

All the patients who were eligible according to the inclusion and exclusion criteria agreed to attend this study and signed the informed consent form (ICF), thereby indicating their full understanding of the study, as well as their rights to confidentiality and withdrawal from participation without providing a reason.

### Study protocol

Patients meeting the inclusion criteria without any exclusion criteria were enrolled within 48 h of CICU admission. Patients with significant pleural effusion at admission could be included following thoracentesis and complete drainage. After obtaining informed consent, baseline demographic data were collected. Within 2 h after enrollment, the ReDS assessment and lung ultrasound examination were performed. The lung ultrasound was conducted first, followed by the ReDS. The results of the ReDS tests were blinded to the physician conducting the LUS examination and result interpretation. Physical examination and measurement of N terminal pro B type natriuretic peptide (NT-proBNP) were all completed on the same day for all of the participants. Other concurrent laboratory parameters, echocardiographic features and medication details were abstracted from the electronic medical record system. The total loop diuretic dose was converted to a furosemide equivalent dose (80 mg furosemide = 40 mg torsemide = 25 mg hydrochlorothiazide), which was based on the study of Levy et al. (17), with hydrochlorothiazide included only when used as an adjunct to loop diuretics.

### Lung ultrasound

Lung ultrasound examinations were performed by trained cardiologist with a portable ultrasound equipment (GE Healthcare, Vivid iq) at the bedside employing a phased array transducer. Based on the international recommendations (18, 19), LUS was recorded in eight thoracic zones (four zones on each hemithorax) at 15–18 cm imaging depth depends on the size of the patient in the semi-recumbent position. Three-second video clips were acquired and stored for offline analysis. Two experienced investigators, blinded to clinical data, examination timing, and patient outcomes, independently interpreted de-identified LUS videos. The maximum number of B-lines visualized within a single intercostal space was recorded for each zone. The total count of B-lines across all eight zones was used for subsequent analyses. A positive indication of pulmonary congestion was defined as the presence of one or more zones exhibiting at least three B-lines on each hemithorax (20).

### ReDS system

**Figure 1:**
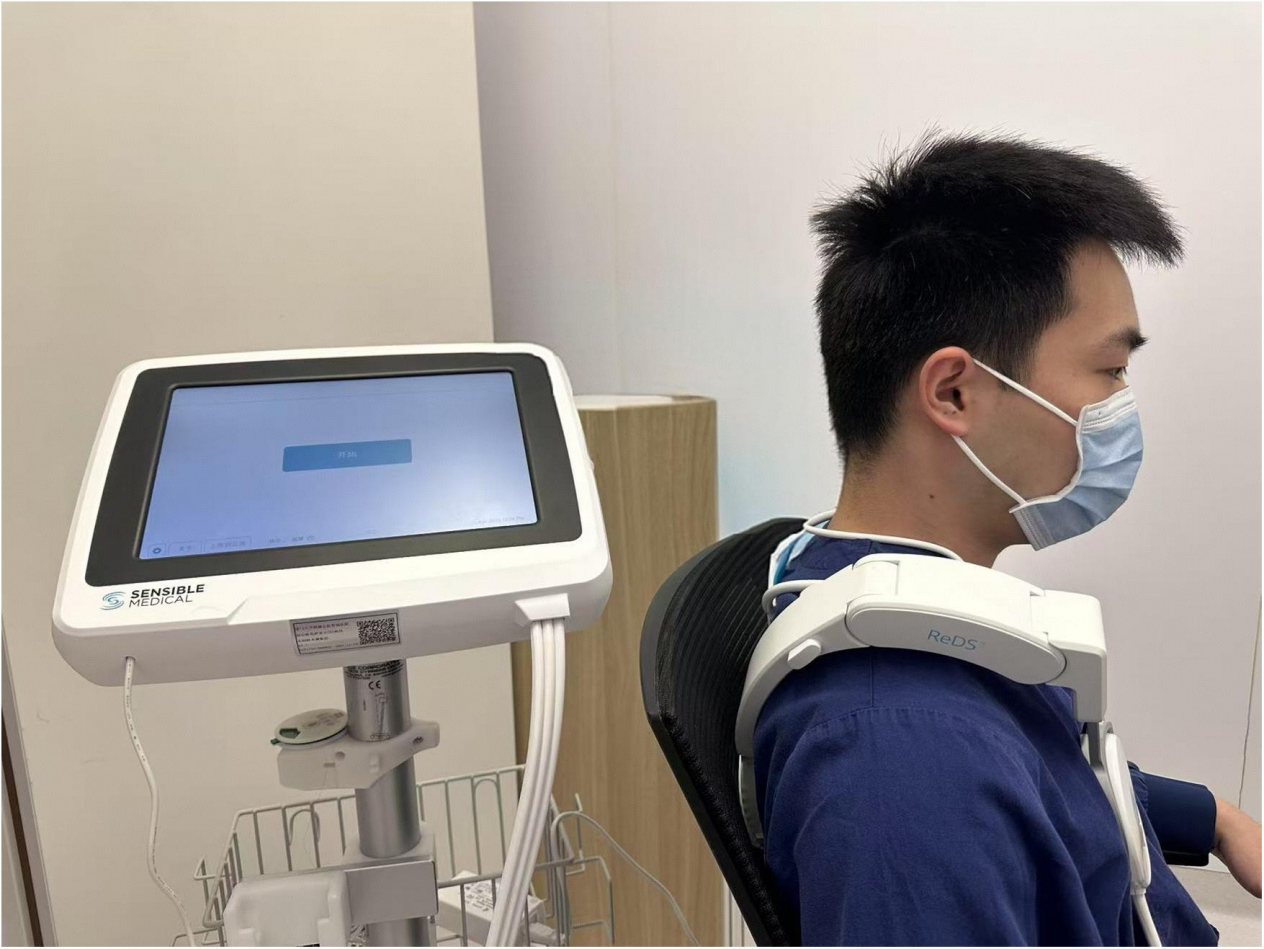
The measurement of remote dielectric sensing system. Figure contains images of the author(s) only.

The Remote Dielectric Sensing (ReDS™, Mitrassist Lifesciences Limited, China) has been extensively described in prior researches (13, 21–23). In brief, ReDS is a system designed to quantify lung fluid content by analyzing the dielectric properties of the lung employing low-power electromagnetic signals transmitted between two sensors. During measurement, patients remained in a seated position while wearing a specialized vest that positioned two sensors on the right hemithorax (Figure 1). Each ReDS assessment required 45 s of signal acquisition following proper vest placement. As specified by the manufacturer, normal ReDS values range from 20% to 35%.

### Statistical analyses

A test for normality of data distribution was conducted using a Shapiro–Wilk test. Normally distributed continuous data was expressed as mean ± standard deviation, while non-normally distributed continuous data was presented as median with an interquartile range. Categorical variables were summarized as frequencies and percentages. We used Mann–Whitney *U* test for comparison of ReDS values between different groups. Correlations were assessed using Spearman's correlation coefficient. Receiver operating characteristic (ROC) curve analysis with Youden's index was performed to identify the optimal ReDS cutoff value for detecting LUS-confirmed pulmonary congestion. For the assessment of association of variables, including ReDS measurement on B-lines, univariate and multivariate linear regression analyses were performed. *P*-value less than 0.05 was regarded as statistically significant. All statistics was done with SPSS 22.0 statistical software and figures were drawn with GraphPad Prism 9.2 software for Windows (GraphPad Software, San Diego, California, USA).

## Result

### Clinical characteristics

A total of 176 patients were screened for eligibility in this study. Of these, 153 patients met the inclusion criteria and were included in the final analysis. The remaining 23 patients were excluded for the following reasons: refusal to participate (*n* = 10), endotracheal intubation (*n* = 3), severe underlying pulmonary disease (*n* = 2), low body weight (*n* = 2), extracorporeal membrane oxygenation (ECMO) implantation (*n* = 1), massive pleural effusion (*n* = 1), and right-sided pacemaker implantation (*n* = 1). The detailed clinical characteristics of enrolled patients were outlined in Table 1. Median age was 74 (60, 81) years old and median body mass index was 22.9(20.5, 25.7) kg/m^2^. The majority of patients were male (60.1%) and had a preadmission history of hypertension (58.8%) and chronic kidney disease (51.6%). The study cohort comprised patients with varying left ventricular ejection fraction (LVEF) ranges, demonstrating a median LVEF of 42(34, 56) %. Concomitant valvular regurgitation was prevalent, with mitral regurgitation present in 82.4% of cases and tricuspid regurgitation observed in 64.1% of the population. Median level of NT-proBNP was 6,891.2(2,123, 20,503.5) pg/mL while the median creatinine was 119.3(88.3, 170.9) umol/L. On physical examination, bilateral pulmonary moist rales were present in 28.8% of patients, while 34.6% exhibited bilateral lower extremities edema. At enrollment, 81% of the patients was receiving loop diuretic therapy, median dose of diuretics was 60(10, 100) mg, and the time from diuretics use to ReDS assessment was 22(0, 29) h.

**Table 1:** Clinical characteristics.

Characteristics	*N* = 153
Demographics	
Age, years	74 (60, 81)
Men, *n* (%)	92 (60.1)
Body mass index, kg/m^2^	22.9 (20.5, 25.7)
Smoking, *n* (%)	34 (22.2)
Comorbidity	
Atrial fibrillation, *n* (%)	53 (34.6)
Diabetes mellitus, *n* (%)	66 (43.1)
Hypertension, *n* (%)	90 (58.8)
Chronic kidney disease, *n* (%)	79 (51.6)
Classification of heart failure	
* *Heart failure with reduced ejection fraction, *n* (%)	58 (37.9)
* *Heart failure with mildly reduced ejection fraction, *n* (%)	42 (27.5)
* *Heart failure with preserved ejection fraction, *n* (%)	53 (34.6)
Echocardiographic parameters	
Left ventricular end-diastolic diameter, mm	51 (46, 59)
Left ventricular ejection fraction, %	42 (34, 56)
Left atrial diameter, mm	42 (38, 47)
Pulmonary hypertension, *n* (%)	54 (35.2)
* *Mitral regurgitation, *n* (%)	126 (82.4)
* *Aortic valve regurgitation, *n* (%)	69 (45.1)
* *Tricuspid regurgitation, *n* (%)	98 (64.1)
Laboratory data	
* *White blood cell, ×10^9^/L	8.4 (6.7, 11.4)
* *Hemoglobin, g/L	118.3 ± 28.3
* *NT-ProBNP, pg/mL	6,891.2 (2,123, 20,503.5)
* *Serum albumin, g/L	35.6 ± 4.2
Alanine Aminotransferase, U/L	23 (14.5, 41.1)
Aspartate aminotransferase, U/L	33.4 (22.9, 60.5)
Serum creatinine, umol/L	119.3 (88.3, 170.9)
Estimated glomerular filtration rate, mL/min/1.73 m^2^	48.1 (28.5, 70)
Physical Examination	
* *Bilateral pulmonary moist rales, *n* (%)	44 (28.8)
* *Bilateral lower limb edema, *n* (%)	53 (34.6)
Medications	
Beta-blocker, *n* (%)	131 (85.6)
Mineralocorticoid receptor antagonist, *n* (%)	83 (54.2)
Renin–angiotensin–aldosterone inhibitor, *n* (%)	72 (47.1)
Sodium-glucose cotransporter 2 inhibitor, *n* (%)	90 (58.8)
Loop diuretics, *n* (%)	124 (81)
Dose of diuretics (mg)	22 (0, 29)
Time from diuretics use to examinations(h)	60 (10, 100)
LUS examination	
Sum of B-lines	7 (2.5, 12.5)
Pulmonary congestion positive, *n* (%)	49(32)
ReDS, %	32(27, 38)

NT-proBNP, N terminal pro B type natriuretic peptide; LUS, lung ultrasound; ReDS, remote dielectric sensing.

**Figure 2:**
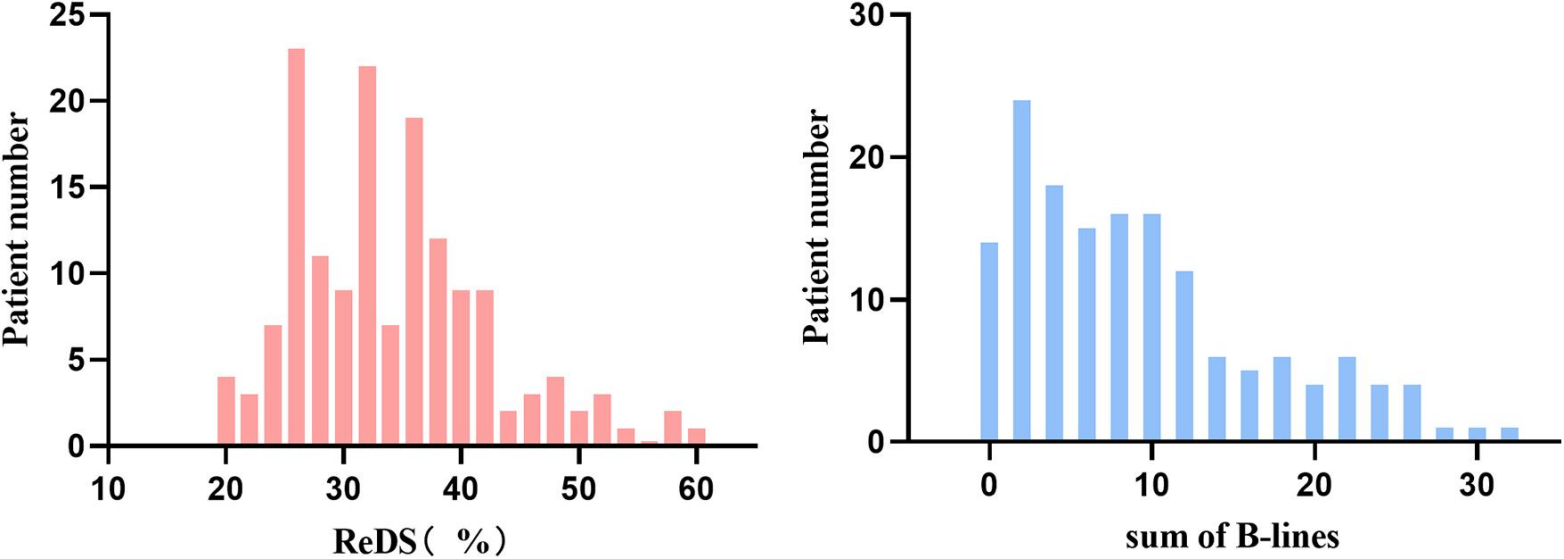
Distributions of remote dielectric sensing (ReDS) and sum of B-lines.

Median values were 32% (range 16%–59%) for ReDS measurements and 7 (range 0–32) for B-lines count. Pulmonary congestion, as defined by LUS criteria, was presented in 32% (*n* = 49) of the study population. The distribution of ReDS values and sum of B-lines were presented in Figure 2.

### ReDS values in different groups

**Figure 3:**
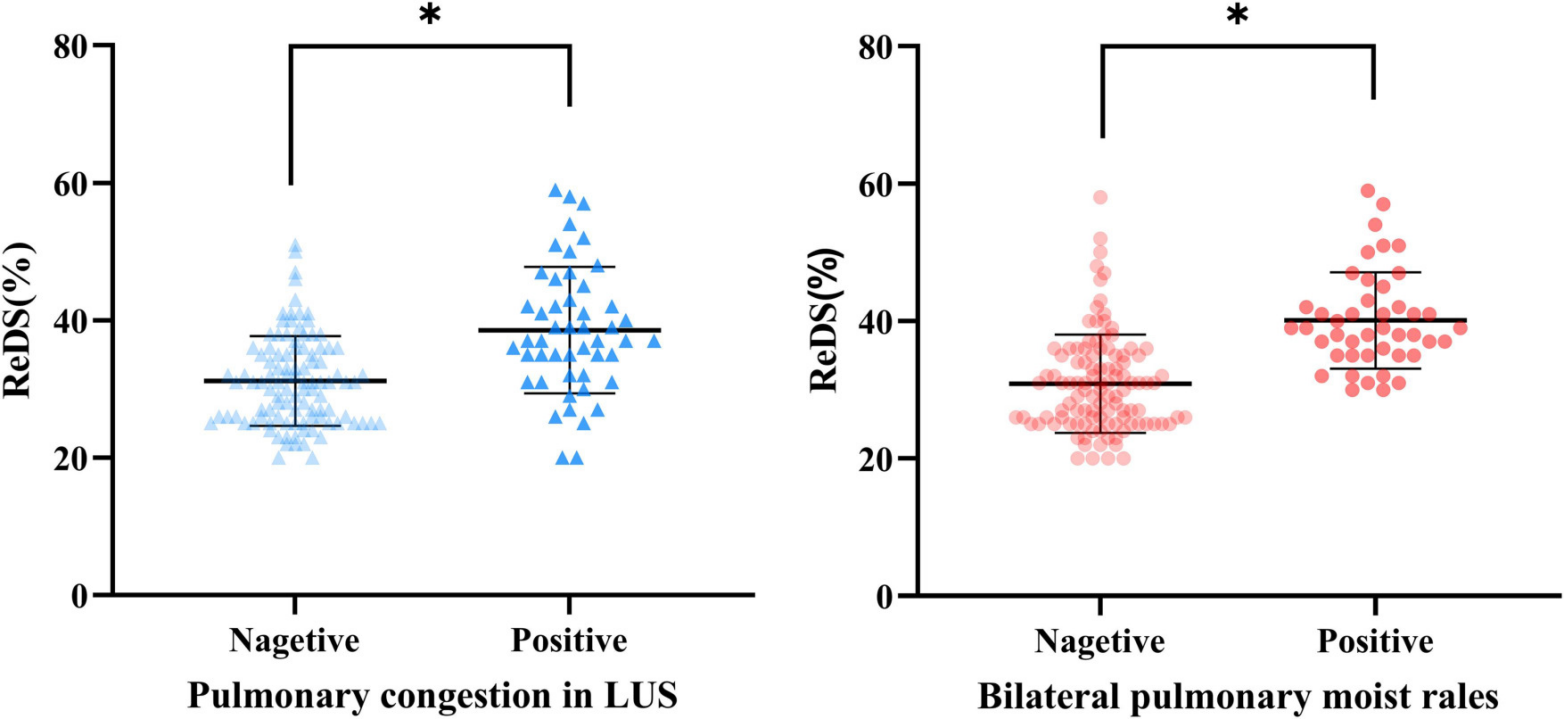
Remote dielectric sensing (ReDS) levels at each group. *P < 0.001; ns, no significance.

Patients with significant pulmonary congestion on LUS demonstrated significantly higher ReDS values than those without congestion (37% vs. 31%, *p* < 0.001). Similarly, patients with bilateral pulmonary moist rales on physical examination showed higher ReDS values compared to those without rales (39% vs. 30%, *p* < 0.001) (Figure 3).

### Correlation analysis

**Figure 4:**
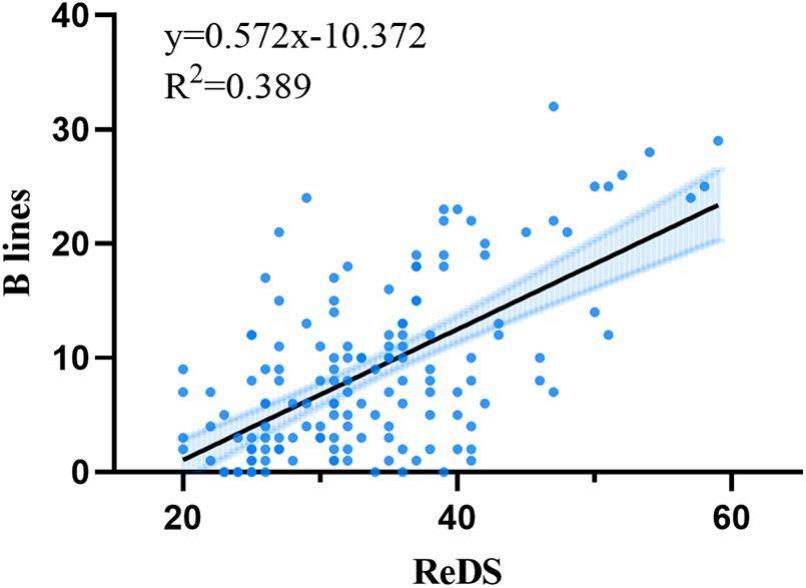
Relationships between remote dielectric sensing (ReDS) and sum of B-lines.

Spearman correlation analysis revealed a significant positive correlation between ReDS measurements and the sum of B-lines (*r* = 0.544, *p* < 0.001). There was no significant correlation between ReDS value and NT-ProBNP, left ventricular end-diastolic diameter, left ventricular ejection fraction, left atrial diameter, creatinine and estimated glomerular filtration rate (Table 2). The linear regression analysis yielded the following equation: sum of B-lines = 0.572ReDS-10.372 (Figure 4).

**Table 2:** Correlations between ReDS level and continuous variables.

Variables	Spearman's *r*	*P*-value
Sum of B lines	0.544	<0.001*
NT-ProBNP, pg/mL	0.007	0.934
LVEDD, mm	0.152	0.061
LVEF, %	0.035	0.669
Left atrial diameter, mm	0.115	0.155
Creatinine, umol/L	0.026	0.745
eGFR, mL/min/1.73 m^2^	0.057	0.480

NT-proBNP, N terminal pro B type natriuretic peptide; LVEDD, left ventricular end-diastolic diameter; LVEF, left ventricular ejection fraction; eGFR, estimated glomerular filtration rate.

*, *p* < 0.05.

Univariate linear regression analysis identified potential variables associated with B-lines count. In the subsequent multivariate model, which adjusted for these potential confounders, both ReDS values (*β* = 0.569, *p* < 0.001) and NT-proBNP levels (*β* = 1.424, p < 0.001) emerged as strong and independent predictors of the B-lines count (Table 3). Ln-transformed NT-proBNP was used in regression models.

**Table 3:** Univariate and multivariate linear regression analysis for sum of B lines.

Variables	Univariate Analysis		Multivariate Analysis	
	Beta Value (95% CI)	*p* Value	Beta Value (95% CI)	*p* Value
ReDS, %	0.572 (0.457–0.687)	<0.001*	0.569 (0.458–0.680)	<0.001*
NT-ProBNP, pg/mL	1.416 (0.604–2.229)	<0.001*	1.424 (0.721–2.128)	<0.001*
BMI, kg/m^2^	−0.188 (−0.475–0.098)	0.196	-	-
LVEDD, mm	0.061 (−0.050–0.172)	0.279	-	-
Creatinine, umol/L	0.006 (−0.001–0.012)	0.09	−0.002(−0.008–0.003)	0.383
LVEF, %	0.021 (−0.068–0.109)	0.645	-	-

NT-ProBNP was modeled as Ln-transformed continuous variable. ReDS, remote dielectric sensing, BMI, body mass index; LVEDD, left ventricular end-diastolic diameter; LVEF, left ventricular ejection fraction.

*, *p* < 0.05.

### ROC analysis

**Figure 5:**
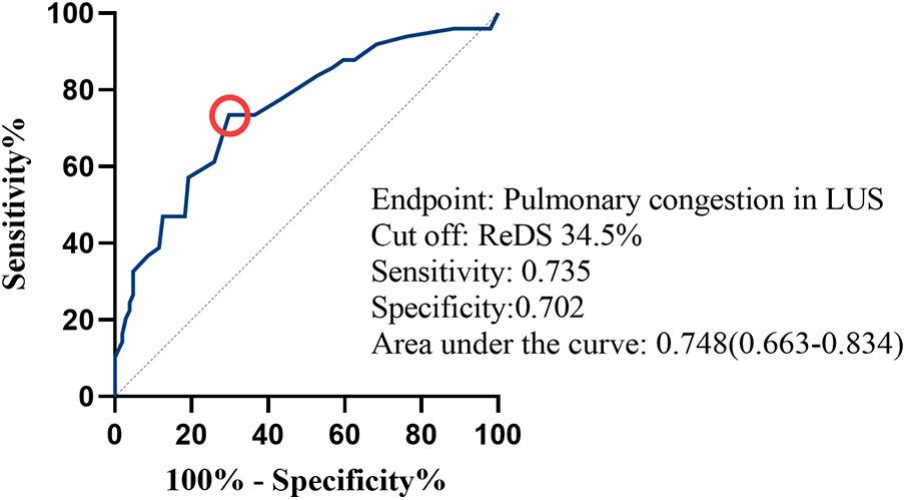
Receiver operating characteristics analysis for remote dielectric sensing (ReDS) values to estimate pulmonary congestion on lung ultrasound (LUS). A red circle indicates the cut-point of ReDS value.

In receiver operating characteristics analysis, the area under the curve of ReDS for predicting pulmonary congestion on LUS was 0.748[95% CI (0.663–0.834), *P* < 0.001]. The optimal ReDS cut-of value for prediction was 34.5% with 73.5% sensitivity and 70.2% specificity (Figure 5).

## Discussion

In the present study, we investigated the association between ReDS and LUS for assessing lung fluid levels in hospitalized patients with heart failure. A statistically significant, moderate correlation was found between ReDS values and the total number of B-lines. Furthermore, ReDS showed fair diagnostic performance for identifying pulmonary congestion, suggesting it may serve as a useful assessment tool in volume management. While previous studies have also explored the relationship between ReDS and LUS, such as the work by Izumida et al. (24), which reported acceptable predictability of ReDS in identifying significant pulmonary congestion in a small cohort of 19 heart failure patients, and Nielsen et al. (22), who found that ReDS performed similarly to LUS in detecting acute heart failure among 97 emergency department patients, the relationship between LUS and ReDS has not been well defined across broader patient populations and more diverse clinical settings. Our study, with a larger and more varied patient sample, extends these earlier observations by supporting the independent association between ReDS and B-lines, and by establishing a cutoff value with satisfactory diagnostic accuracy. This adds to the growing body of evidence supporting the use of ReDS in heart failure management, particularly in quantifying lung fluid content and potentially complementing LUS in clinical practice.

Volume management is a major focus in the treatment of heart failure, aimed at achieving and maintaining a state of euvolemia. The escalating signs and symptoms of congestion are the primary factors prompting patients with acute heart failure to seek urgent medical attention which are linked to elevated morbidity and mortality rates, thereby imposing a significant economic burden on our society (2, 25). In this regard, assessment tools focused on the measurement of fluid status is essential to individualize and dynamically adjust therapeutic strategies, and to keep the stability of the patients (26). Although invasive cardiac catheterization is considered the gold standard for quantifying congestion, its procedural risks preclude routine clinical use (27, 28). Clinical signs and conventional imaging often fail to detect subclinical or early-stage congestion, leading to its frequent underestimation by clinicians. This diagnostic gap can result in inadequate decongestive therapy and poor patient outcomes (29). Advancing innovative, precise, non-invasive, and safe measurement techniques remains a top priority. A successful HF monitoring tool should meet at least five prerequisites: appropriate signal, accurate, absolute values, actionable and algorithm (30). ReDS is a recently introduced technology that fulfills five essential prerequisites, thereby emerging as a promising clinical tool. LUS is a non-invasive and versatile point-of-care diagnostic tool that is valuable for the diagnosis, monitoring, and prognostic assessment of HF patients. Due to its reliability and clinical utility, LUS has been established as a standard modality for assessing pulmonary congestion (6). Thereby, this study validated the capacity of ReDS systems to quantify pulmonary congestion through consistency analyses with LUS.

In LUS assessments, an eight-zone scanning protocol was adopted to achieve optimal balance between examination efficiency and accuracy, with B-lines quantified using a count-based method. We demonstrated a positive correlation between the ReDS value and total number of B-lines. In fact, a perfect correlation between ReDS and LUS was not expected. The total B-line count was obtained through evaluation of eight bilateral chest regions. In contrast, ReDS measurements were acquired from the right hemithorax. The distribution of extravascular lung water in certain patient populations may demonstrate asymmetry between the bilateral lungs, influenced by inherent anatomical disparities, comorbid respiratory conditions, and hemodynamic variability (31). Moreover, ReDS provides a continuous, direct estimate of fluid volume, whereas LUS provides a discrete, ordinal count that, despite being quantitative, does not have a strictly linear relationship with absolute fluid volume. The above reasons may contribute to some discordance between these modalities.

Lung ultrasound also enables qualitative assessment of pulmonary congestion through analysis of B-line patterns. Our analysis demonstrated that ReDS technology achieved 75% accuracy in detecting pulmonary congestion on LUS with a cutoff value of 34.5%. This finding aligns with Nielsen et al.'s report of 71% accuracy for ReDS in identifying acute heart failure among patients with dyspnea (22). The selected cutoff value corresponds with previous research by Abbo et al., who established that ReDS values below 34% exhibited high negative predictive value for excluding patients with pulmonary artery wedge pressure (PAWP) > 17 mmHg in heart failure populations (13), while also being consistent with the manufacturer's recommended normal range. The ideal threshold may vary slightly around 35% depending on the clinical context and comparator. However, the optimal diagnostic threshold for ReDS may not be universally constant, with literature suggesting variability potentially attributable to patient physique. This was exemplified by a study where a lower cutoff of 28% was optimal in a cohort with a high prevalence (57%) of patients with small stature (body height <155 cm) (23), Furthermore, recent evidence indicates a systematic underestimation of ReDS values in underweight individuals, who presented with the lowest values across BMI categories (32). These consistent findings across different physique extremes suggested that the current ReDS algorithm may not be fully optimized for all body habitus. Consequently, future research should focus on developing physique-specific interpretive frameworks or refined calibrations to ensure accurate device performance across the spectrum of patient demographics, particularly for underweight and small-stature populations.

Physical examination remains one of the most accessible clinical methods for assessing volume status. In patients with heart failure, bilateral pulmonary moist rales serve as characteristic auscultatory findings indicative of pulmonary congestion. Our study demonstrated that patients exhibiting bilateral pulmonary moist rales had significantly higher REDS values compared to those without this physical finding. Additionally, approximately 30% of patients with unremarkable pulmonary auscultation results presented with ReDS values ≥35%, which aligns with the recognized limitations in sensitivity of physical examination for detecting pulmonary rales. These findings illustrate how the quantitative assessment by ReDS relates to traditional clinical evaluation, highlighting its potential complementary role in detecting pulmonary congestion.

Natriuretic peptides (NPs), cardioprotective hormones secreted by cardiomyocytes in response to pressure or volume overload, are well-established biomarkers for excluding acute heart failure due to their high negative predictive value (33, 34). Beyond diagnostic utility, NPs also play a crucial role in risk stratification for HF patients (35). Contrary to previous reports by Kinugawa et al. (21), our study did not demonstrate a significant correlation between ReDS values and NT-proBNP levels. This discrepancy may be attributable to several confounding factors influencing NT-proBNP concentrations, including advanced age, sex differences, and renal impairment (36). Notably, our cohort had a median age of 74 years, and over 50% of participants exhibited renal insufficiency, which made NT-proBNP unable to serve as an isolated indicator of volume status in such populations.

The ReDS system represents a novel technology that can accurately quantify changes in lung fluid concentration noninvasively, offering distinct advantages over other available modalities for quantifying extravascular lung water. Its clinical utility stems from three key characteristics: (1) portability enabling use across diverse settings (hospitals, communities, and home environments), (2) operational simplicity allowing administration by healthcare professionals or caregivers, and (3) objective, quantitative output minimizing interpreter variability. Growing evidences supported the clinical application value of ReDS monitoring in heart failure management. It has been reported that the utilization of ReDS technology for monitoring pulmonary congestion during hospitalization may serve as a significant prognostic marker for outcomes in HF populations (37). Findings from retrospective studies indicated that ReDS-guided volume management, when implemented during both inpatient care and the post-discharge transition period, may contribute to improved clinical outcomes in cardiovascular patients (29, 38). Most compellingly, A recent randomized clinical trial revealed that a ReDS-guided decongestion strategy significantly improved 30-day post-discharge outcomes in acute decompensated heart failure patients (39). Building on the present findings and existing literature, a clearer perspective on the integration of ReDS into clinical workflows emerges, suggesting that employing it for precise lung fluid assessment to guide tailored volume management, both during hospitalization and after discharge, holds promise for improving patient outcomes.

In clinical practice, both ReDS and LUS are valuable surrogate measures with complementary strengths and limitations. LUS offers the advantage of potentially aiding in differential diagnosis and is well-suited for critically ill, bedridden patients. In contrast, ReDS provides a rapid, operator-independent quantitative assessment without requiring patient disrobing, making it particularly suitable for serial measurements not only in hospitals but also in outpatient and community settings post-discharge. Therefore, based on our results demonstrating concordance with LUS, ReDS could serve as a viable alternative or adjunct to LUS. Ultimately, the choice between these modalities can be guided by specific clinical needs and available resources, with the shared goal of enabling accurate congestion monitoring to guide effective decongestive therapy. Further studies are warranted to establish standardized protocols for its implementation in combined assessment strategies.

The current study has several limitations that should be acknowledged. First, as a single-center investigation, the sample was derived from a single institution, which may restrict the generalizability of the findings to broader populations due to potential selection bias and institutional-specific clinical practices. Meanwhile, as an exploratory study investigating the correlation between ReDS and LUS, a formal pre-study sample size calculation was not performed. The enrollment target was set based on feasibility and was comparable to the sample sizes of prior pilot studies assessing the ReDS technology (13, 23). Second, since pleural effusion interfered with B-line assessment, we excluded patients with significant effusion. Consequently, the potential influence of pleural effusion on ReDS values remains unexplored, which may limit the generalizability of our findings to real-world patients who present with mixed patterns of congestion. Further studies are needed to investigate the specific impact of pleural effusions on ReDS measurements and to develop integrated assessment models. Third, we excluded critically ill patients receiving mechanical ventilation, IABP, or ECMO. Due to the positional restrictions during the detection process, the application of REDS in the assessment of such critically ill patients was restricted. In addition, our study excluded patients with severe pulmonary comorbidities to minimize potential confounders in establishing the ReDS-LUS relationship. This may limit the direct generalizability of our findings to heart failure populations with overlapping lung diseases. This consideration is particularly relevant in light of recent evidence suggesting that pulmonary conditions can influence ReDS measurements (40). Future studies should therefore focus on validating these findings in more diverse, real-world patient populations that include individuals with concurrent cardiac and pulmonary conditions. Finally, our study focused exclusively on hospitalized patients. Whether these findings extend to remote monitoring or home care settings remains unclear and should be addressed in subsequent research.

## Conclusion

In this study, A significant correlation was observed between ReDS-measured and LUS-assessed extravascular lung fluid in heart failure patients. This finding, along with the fair diagnostic accuracy of ReDS, lends support to its potential role in the clinical evaluation of pulmonary congestion. Further research is needed to explore broader applications of ReDS and establish standardized protocols for its implementation in clinical practice.

## Data Availability

The raw data supporting the conclusions of this article will be made available by the authors, without undue reservation.
